# Falls and balance impairment; what and how has this been measured in adults with joint hypermobility? A scoping review

**DOI:** 10.1186/s12891-025-08318-3

**Published:** 2025-01-28

**Authors:** Yiduo Wang, Paul H. Strutton, Caroline M. Alexander

**Affiliations:** 1 The Nick Davey Laboratory, Division of Surgery, Department of Surgery and Cancer, Faculty of Medicine, Sir Michael Uren Hub, Imperial College London, White City Campus, 86 Wood Lane, London, W12 0BZ UK; 2 Department of Therapies, Charing Cross Hospital, Imperial College Healthcare NHS Trust, Fulham Palace Road, London, W6 8RF UK

**Keywords:** Falls, Joint Hypermobility, Balance

## Abstract

**Background:**

People with joint hypermobility have excessive joint flexibility, which is more common in young women. The people with symptomatic hypermobility report poor balance and even falls. This scoping review aims to identify and map the available evidence related to balance and falling in adults with joint hypermobility to support research planning and ideas for treatment direction.

**Methods:**

A framework for the search was constructed using the Joanna Briggs Institute (JBI) approach. Electronic searches of primary evidence were performed using the following databases: Medline, Web of Science, CINAHL, Scopus and EMBASE. Papers written in English and published between 1946 and November 2023 were included. Titles, abstracts, and full papers were independently screened by two reviewers. Data extracted related to the population, the concept under investigation, the method of measurement, the level of evidence and the result.

**Results:**

Nineteen of 1,950 screened articles were included. In terms of the population, details related to ethnicity of the research participants was missing. The hypermobility classification criteria varied; it was not always clear if people who were hypermobile had symptoms. Concepts related to fear of falling, balance and adaptive strategies prompted by different postural tasks with and without vision were explored. Centre of pressure, muscle activity, kinematics and psychological factors were measured using force plates, electromyography (EMG), motion capture, patient and clinician reported outcome measures, focus groups and interviews. Most papers were low on the hierarchy of evidence (i.e. equal or lower than a case control study). The participants with joint hypermobility had increased sway, different muscle activity, and different kinematics compared to people without hypermobility. When surveyed, they commonly fell and had a fear of falling.

**Conclusions:**

It is unclear whether the participants represent the population of people with hypermobility. Different classification systems were used making it difficult to generalise the results. Although the methods used suggest a low level of evidence, it seems clear that people who are hypermobile have poor balance. The underlying mechanisms driving poor balance have not been explored in depth.

**Supplementary Information:**

The online version contains supplementary material available at 10.1186/s12891-025-08318-3.

## Background

Joint hypermobility is a condition where one or more joints move beyond their normal range of motion [1]. The prevalence of joint hypermobility in adults is not clear but may range between 12.5% to 38% [2, 3]; this varies depending on age, sex, and ethnicity [4, 5]. During aging, joint hypermobility declines due to the natural increase in body stiffness [6]. It is also more common in females [7], Asians, Africans and Middle Eastern descent [8, 9]. With the high prevalence in different populations, it is important to understand the impact of the condition.

Joint hypermobility can be asymptomatic or symptomatic. Generalised joint hypermobility (GJH) was a term used to describe the asymptomatic population [1]. Here, individuals only exhibit joint hypermobility without accompanying symptoms [1]. On the one hand, people with asymptomatic hypermobility may have a greater risk of musculoskeletal injury [10], but on the other hand it may confer benefits under certain circumstances, for example for people involved in classical dance [11] and certain sports [12] where they may gain advantage through the extra range of motion. A small proportion of people with joint hypermobility suffer with symptoms such as pain, joint instability, altered motor control and poor balance [7]. Where genetic tests are negative for conditions of hypermobility, such as Marfan syndrome and other types of Ehlers-Danlos Syndrome (EDS), there is no gold standard test for classifying the majority of people with symptomatic joint hypermobility. Further, the names and classification systems for people without genetic markers have changed over time [13–16]. In 2017, these people with symptomatic joint hypermobility have been classified as having hypermobile spectrum disorder (HSD) or hypermobile Ehlers-Danlos Syndrome (hEDS) [1].

Despite uncertainty over classification systems, it is clear that people are asymptomatic or symptomatic, and when symptomatic they suffer a broad range of symptoms which impact their function and quality of life [17].

One symptom suffered is poor balance, which can lead to falls [18, 19] i.e. when someone comes “to rest inadvertently on the ground or floor or other lower level” [20]. Although studies often focus on balance and falls in an elderly population, the scope of the evidence related to balance and falls in people with hypermobility is unclear. Yet, this is important as it has previously been identified that 95.5% of people with hypermobility, self-report that they have fallen at least once per year, and at worst, more than once per week [21]. This reduced balance could be related to many factors such as muscle weakness [22], poor proprioception [23] as well as postural orthostatic tachycardia (POTs) [24, 25]. However, there is not sufficient quality data to justify a systematic review to understand the mechanisms which might underlie this poor balance. The purpose of this study is to understand the scope of the evidence related to balance and falls in adults with joint hypermobility, which will help to direct research planning towards the gaps in this evidence.

Our questions included:

1. Are adults with hypermobility recruited to studies investigating balance and falls representative of their population?
2. How was the hypermobility of participants classified?
3. What is the level of the evidence published?
4. What factors related to balance and falls have been investigated?
5. How have they been measured and in what context?
6. What are the results of these studies?

## Methods

The scoping review was conducted in accordance with the population, concept and context (PCC) methodology recommended by the Joanna Briggs Institute (JBI) Framework and reported following the PRISMA-ScR framework for scoping reviews (see additional file 2 PRISMA-ScR Checklist) [26, 27]. Our patient and public involvement and engagement (PPIE) group were 6 people with lived experience of symptomatic hypermobility. They either work within the university sector or have contributed to previous research projects related to hypermobility. They supported the direction of our research questions and the construction of the review by suggesting we explore factors, which they report increase their risks of falling or lead to imbalance (e.g. pain, POTs, poor proprioception, dizziness, anxiety, fatigue) and refining the research strategy.

### Inclusion and exclusion criteria

Papers were included if published in English, were primary evidence and recruited adults aged between 18 to 65. Our search terms ensured we included participants with symptomatic and/or asymptomatic joint hypermobility using terms such as “joint hypermobility” and “hypermobility Ehlers Danlos Syndrome” (see additional file 1 Search Strategy). In addition, they investigated balance and falls, using methods and outcomes which relate to balance and falls. Papers were excluded if participants had genetically identifiable heritable connective tissue disorders related to hypermobility e.g. Marfan syndrome or genetically identifiable EDS sub-types such as classic EDS or vascular EDS. In addition, papers were excluded if they related to people with hypermobility of a single joint due to injury or surgery. Reviews, single case studies, author opinion pieces and conference proceedings were also excluded.

### Search strategy

The search of electronic databases included Medline, Web of Science, CINAHL, Scopus, and EMBASE and was undertaken on January 20th, 2023. The search was updated on November 15th, 2023. Papers written in English and published between 1946 and November 2023 were included. The search terms were developed by the authors and the PPIE group. Keywords were gathered through brainstorming, by exploring keywords of relevant papers, as well as using appropriate Medical Subject Headings. The full search strategy is within the supplementary material (see additional file 1 Search Strategy) but included hypermobility, Ehlers-Danlos Syndromes with the concept such as falls and balance and outcomes and methods related to the exploration of balance and falls such as electromyography (EMG), vestibular function tests, force plate, and Berg balance scale. The search strategy, with the appropriate truncation was adapted for each database. The reference lists of all papers included in the final list were screened for additional studies.

Following the search, all identified citations were collated and uploaded into EndNote 20 (Clarivate Analytics, PA, USA) and duplicates removed. The data were then imported into Covidence 2023 System (Covidence, Melbourne, Australia) to allow for efficient data management. Titles and abstracts were screened independently by two reviewers (YW and CMA) for assessment against the inclusion criteria. Reasons for exclusion were recorded at full text stage. Any disagreements that arose between the reviewers at each stage of the selection process were resolved through discussion and if consensus couldn’t be reached the opinion of a third reviewer (PHS) would be sought.

### Data extraction

The included papers were reviewed by two authors (YW and CMA); some papers were further excluded as, on reading the full text, it became clear they did not fulfil the inclusion criteria. Data were extracted using a bespoke data extraction table, developed after piloting, which followed the Population Concept Context (PCC) template [26]. Any disagreements that arose between the reviewers were first discussed, and if it had been needed, would have been resolved through discussion with the third reviewer (PHS). Data extracted included relevant population details such as participant demographics (i.e. sex, age, ethnicity) and hypermobility classification criteria; the concepts (i.e. the topic of the research, such as falling, balance, postural sway/instability) and the context (i.e. year of publication, country of origin, research setting). Details related to study methods were extracted (i.e. sample size, study design, interventions, outcome measures and level of evidence [25]. Levels of evidence were determined using an evidence-based medicine pyramid [28]. Finally, key findings relevant to the review questions were recorded. An assessment of quality or risk of bias was not undertaken as our aim was to provide an overview of the evidence [27]. Likewise, the nature of the studies available for review meant quality assessment would not meaningfully enhance interpretation or discussion of findings. Instead of that, levels of evidence were detailed labelling each paper.

## Results

**Fig. 1:**
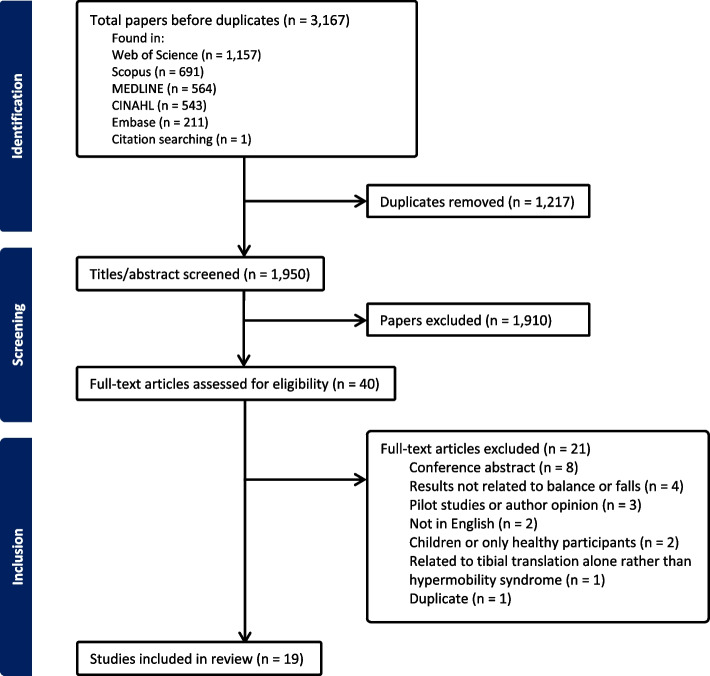
PRISMA Diagram to illustrate the flow of papers from initial screening to inclusion

Figure 1 displays our process for the selection of evidence. A total of 3167 papers were retrieved across all electronic databases. After duplicates were removed, 1950 articles were included in the review. Once the titles and abstracts were screened, 40 articles were eligible for a full-text review. Following this, 19 articles were identified as being appropriate for inclusion.

### Population

The majority of the papers related to young, adult women, which is detailed in Table 1. Importantly, classification of hypermobility varied, and it was not always clear if the population were symptomatic or asymptomatic as the Beighton score was often used alone, rather in combination with other systems such as the Brighton [15] or that for Hypermobility Spectrum Disorder [29]. In addition, even when the Beighton score was used, authors had different cut off points to define whether someone was hypermobile or not [7, 21, 30, 31]. A key finding was that some protected characteristics described by the UK Equality Act of 2010 [32], such as ethnicities were rarely recorded (see Table 1) [33].

**Table 1:** Information related to participant characteristics of research subjects

		Symptomatic hypermobility population				Asymptomatic hypermobility population				Comparator population			
Author (year)	Classification criteria for hypermobility	Sex	Age	Ethnicity	Sample Size	Sex	Age	Ethnicity	Sample Size	Sex	Age	Ethnicity	Sample Size
		(Female Proportion)	(mean, median with standard deviation or interquartile range)			(Female Proportions)	(mean or median ± standard deviation or interquartile range)			(Female Proportion)	(mean, median variability)		
Aydın E et al. (2017) [34]	Beighton score	N/A	N/A	N/A	N/A	Not mentioned	21.2 ± 2.8 (Beighton score: 3 - 4)	Not mentioned	13 (Beighton score: 3 - 4)	Not mentioned	21.6 ± 3.4	Not mentioned	29
	(With an addition of cut offs: 0 to 2 not hypermobile, 3 to 4 moderately hypermobile, with 5-9 "distinctly" hypermobile)						20.8 ± 2.9 (Beighton score: 5 - 9)		27 (Beighton score: 5 - 9)				
Falkerslev S et al. (2013) [35]	Beighton score (≥4/9)	N/A	N/A	N/A	N/A	Not mentioned	Median 39.64 (32–51)	Not mentioned	18	Not mentioned	Median 40.09 (31 – 47)	Not mentioned	18
Toprak Celenay S et al. (2017) [36]	Brighton criteria	100%	20.3±2.2 (intervention group) and 21.0 ± 2.2 (control group)	Not mentioned	20 (intervention group) and 18 (control group)	N/A	N/A	N/A	N/A	N/A	N/A	N/A	N/A
Rombaut L et al. (2011) [ 21]	Villefranche criteria	100%	39 ± 10.6	White	22	N/A	N/A	N/A	N/A	100%	39 ± 10.5	White	22
Ulus Y et al. (2013) [37]	Beighton score (≥4/9)	100%	42.2 ± 10.42	Not mentioned	30 (FMS with asymptomatic hypermobility)	100%	42.43 ± 10.52	Not mentioned	30 (FMS without asymptomatic hypermobility)	100%	40.67 ± 9.03	Not mentioned	30
Russek L et al. (2014) [38]	5-part question (GJH)	N/A	N/A	N/A	N/A	97.60%	Median not recorded, the most common age group was between 40–49	Not mentioned	524 (FMS with asymptomatic hypermobility)	Not mentioned	Not mentioned	Not mentioned	N/A
Bates AV et al. (2021a) [39]	Beighton score (≥4/9); Brighton criteria	85.71%	33.0 ± 9.0	Not mentioned	21	84.85%	28.0 ± 6.0	Not mentioned	23	72.72%	28.0 ± 5.0	Not mentioned	22
Iatridou K et al. (2014) [18]	Revised Brighton criteria	100%	21.7 ± 1.7	White	21	N/A	N/A	N/A	N/A	100%	21.5 ± 1.7	White	20
	(cut off ≥4/9 Beighton as major criteria with at least 2 minor criteria, in the absence of genetically tested for other heritable connective tissue disorders)												
Rigoldi C et al. (2013) [40]	Revised Brighton criteria	Not mentioned	32.4 ± 8.4	Not mentioned	13	N/A	N/A	N/A	N/A	Not mentioned	31.4 ± 9.6	Not mentioned	20
	(In the absence of genetically tested for other heritable connective tissue disorders)												
Marnili T et al. (2017) [30]	Beighton-Horan joint mobility index	N/A	N/A	N/A	N/A	Not mentioned	Not mentioned	Not mentioned	135	N/A	N/A	N/A	N/A
	(Beighton without hip flexion therefore out of 8, cut off ≥4/8)												
Galli M et al. (2011b) [41]	Villefranche criteria;	86%	36.8 ± 12.7	Not mentioned	22	N/A	N/A	N/A	N/A	16.6% (PWS);	34.4 ± 3.7 (PWS);	Not mentioned	11 (PWS);
	Beighton score;									50% (healthy)	31.4 ± 9.6 (healthy)		20 (healthy)
	Brighton criteria												
Galli M et al. (2011a) [42]	Not mentioned	Not mentioned	40.8 ± 11.0	Not mentioned	22	N/A	N/A	N/A	N/A	50%	40.1 ± 4.8	Not mentioned	20
Whitmore M et al. (2023) [43]	hEDS 2017 criteria	94.64%	Not mentioned	Not mentioned	56	N/A	N/A	N/A	N/A	N/A	N/A	N/A	N/A
Teran-Wodzinski P et al. (2023) [7 ]	Self-reported hEDS or generalised HSD	90.00%	18-20: 8%	White or European American 85%	483 (396 analysed)	N/A	N/A	N/A	N/A	N/A	N/A	N/A	N/A
			21-30: 30%	Black of African American 0.3%									
			31-40: 27%	Asian American 1%									
			41-50: 18%	American Indian/Alaska Native 1%									
			51-60: 11%	Mixed (two or more) 8%									
			61-70: 3%	Other 3%									
			>70: 1%	Prefer not to say 2%									
Hou ZC et al. (2023) [44]	Beighton score (cut off ≥4/9);	N/A	N/A	N/A	N/A	55.60%	30.2 ± 3.3	Not mentioned	20 (18 analysed)	58.80%	29.0 ± 3.3	Not mentioned	20(17 analysed)
Hakimi A et al. (2023) [45]	hEDS 2017 criteria	94.73%	45.0±12.0	Not mentioned	19 (18 analysed)	N/A	N/A	N/A	N/A	N/A	N/A	N/A	N/A
Benistan K et al. (2023b) [46]	hEDS 2017 criteria	91.00%	31.9 ± 11.5	Not mentioned	32 (29 analysed)	N/A	N/A	N/A	N/A	N/A	N/A	N/A	N/A
Benistan K et al. (2023a) [47]	hEDS 2017 criteria, and EDS as a whole not including Vascular type	Not mentioned (only mentioned 91% in all EDS types)	Not mentioned (33.1 ± 11.2 in all EDS types)	Not mentioned	76 (61 analysed)	N/A	N/A	N/A	N/A	N/A	N/A	N/A	N/A
Bates AV et al. (2021b) [48]	Beighton score (cut off ≥4/9); Brighton criteria	86.96%	33.0 ± 9.0	Not mentioned	23	82.61%	28.0 ± 6.0	Not mentioned	23	72.73%	28.0 ± 5.0	Not mentioned	22

Legend: *Abbreviations:* *N/A* Not applicable, *EDS* Ehlers Danlos Syndromes, *hEDS* hypermobile Ehlers Danlos Syndrome, *GJH* Generalised Joint Hypermobility, *FMS* Fibromyalgia Syndrome

### Concepts

A variety of concepts was investigated by different authors. Concepts included: frequency of falling, fear of falling, postural stability and pain. Mechanisms underlying balance were probed by challenges of proprioception, vestibular and visual systems. In addition, studies evaluated the effectiveness of interventions such as exercise with or without compression garments on balance [36, 44–47].

Different papers used different methods to measure different factors related to balance. Table 2 describes the methods employed. One such technique, which is common in the exploration of balance in other populations, was the use of force platforms to explore posturography. Here authors used outcomes such as frequency [34], velocity and ranges of sway comparing different tasks that stress balance with eyes open and closed [39–42]. Other methods and outcomes included using video systems to measure changes in joint angles under different conditions [39, 48]. Additionally, strength and functional tasks [36, 37, 44, 45]; different patient reported scales and questionnaires [37, 47] and EMG [39] were used and are all detailed in Table 2.

**Table 2:** Methodology and results of included papers

	Methodological approach	Intervention	Comparators if different	Outcome measures	Key findings
Aydın E et al. (2017) [34]	Postural Stability: Posturography	N/A	N/A	1. Postural sway measured using Fourier index in a scale of rising frequency bands.	1. Higher postural sway frequency in the distinctly hypermobile group compared to the non-hypermobile group in standing with head to the right about 45˚, EC, firm surface; a measure of vestibular stress and elimination of visual system. However, no other positions differed (including head to the left)
				2. General stability was calculated by amount of sway over the four integrated platforms without consideration of individuals’ weight and height.	2. Sway amounts increased in GJH group within head right and head raised backward about 30˚, EC, firm surface indicating cervical and vestibular stress and elimination of the visual system positions but no other positions.
				3. The level of weight distribution was analysed by weight distribution index. The coordination between toes and heels was evaluated by synchronization index scores.	3. Weight distribution and coordination did not differ across groups.
Falkerslev S et al. (2013) [35]	1.Dynamic stability: Kinematics and Kinetics	N/A	N/A	1. Horizontal movement (yaw) and lateral rotations (roll) of the head, shoulders, spine, and pelvis during gait.	1. GJH adults showed decreased lateral stability of the shoulder, lumbar and thoracic trunk whilst walking, but it was not associated with decreased stability of the head in any of the walking conditions
				2. Stability was measured as angular dispersion recorded as angle of displacement in degrees.	2. In both walking conditions, GJH adults displayed reduced lateral stability in the shoulder, lumbar, and thoracic trunk; however, this did not correspond with reduced stability in the head during any of the walking conditions.
Toprak Celenay S et al. (2017) [36]	1. Postural stability: Posturography	Group exercise of 45mins with 5-6 people for 3 days a week for 8 weeks. Warm up and progressed trunk stability strengthening exercises. Easiest in static positions with help of biofeedback, progressed to consciously maintaining trunk stability during dynamic tasks with resistance bands and then to unconscious functional tasks within increasing demand on balance with resistance bands.	N/A	1. Trunk muscle endurance was measured by McGill’s trunk muscle endurance tests: trunk flexor; back extensor; right and left lateral trunk musculature.	1. 8 people were lost to follow up, 3 in the control group and 5 in the hypermobile group. Stability index between groups before and after the intervention did not differ across any condition.
	2. Pain: Visual Analog Scale (VAS) and map			2. Static and dynamic postural stability: static mode EO, static mode EC, dynamic mode EO, and dynamic mode EC- Biodex balance system.	2. Change in stability index after the intervention was greater than change in the control group (median and min-max): intervention 0.1 95% confidential interval (CI) −1.2 to 0.8; control 0.1 (95% CI −1.5 to 1.0); p=0.04
	3. Muscle endurance function: McGill’s trunk muscle endurance tests.	Comparator Intervention: No exercise.		3. Pain: Pain area on body map, pain severity using VAS.	
	3. Intervention: exercise.				
Rombaut L et al. (2011) [ 21]	1. Balance: Posturography.	N/A	N/A	1. In bare feet using force plates and measuring mean sway velocity along the COP path (sway velocity; cm/second), standard deviation of ML and AP COP excursion, 95% ellipse sway area during Clinical test of sensory interaction on balance and tandem stance test (x3 for 30sec each) with arms at side, looking ahead.	1. Sway velocity faster in hEDS and greater distance and area of sway. More variable than controls. Stability deteriorated significantly more in hEDS than control subjects when deprived of visual information.
	2. Gait: Kinetic and kinematics.			2. Gait measured during a single-task condition, i.e., preferred walking (single task), and 2 dual-task conditions, i.e., walking while subtracting 3 backward from 100 (cognitive task) and walking while carrying a tray with glasses (functional task).	2. hEDS group walked with decreased speed, with shorter step length, and with shorter stride length compared to the healthy control group.
	3. Fall frequency and fall circumstances: retrospective recall.			3. Fall frequency and fall circumstances (place, cause, use of assistive devices) during the past year were assessed by retrospective recall.	3. Nearly all patients fell at least once during the past year. More specifically, 2 patients (9.1%) reported falling 1 time/week, 10 patients (45.5%). reported falling 1 time/month, and 9 patients (40.9%) reported falling 1 time/year, whereas only 1 patient (4.5%), and all of the control subjects, reported no falls.
	4. Fear of falling: Questionnaires.			4. Fear of falling while performing everyday activities measured using modified version of the Falls Efficacy Scale (FES).	4. Significantly higher FES scores were found in the hEDS.
Ulus Y et al. (2013) [37]	1. Pain: VAS.	N/A	N/A	1. Pain severity was measured with VAS	1. Pain: FMS with asymptomatic hypermobility no different to FMS without asymptomatic hypermobility group (8/10)
	2. Muscle endurance function and fall: the six-minute walk distance (6MWD) test			2. Functional performance and endurance were measured with the six-minute walk distance test	2. Muscle Endurance: no differences across the 3 groups
	3. Static balance: a one-legged balance test with EO.			3. Static balance was measured by a one-legged balance test with EO.	3. Static balance: single leg stance time is shorter for FMS with asymptomatic hypermobility group in comparison to the other two groups (15 vs 25 vs 30 (with stop at 30sec)
	4. Postural stability: the Berg balance scale (BBS).			4. Postural control was measured by the BBS.	4. Postural stability: differs across groups and FMS with asymptomatic hypermobility worst (50 vs 56 vs 56)
	5. Activity: questionnaires			5. Activity was assessed by Fibromyalgia Impact Questionnaire	5. Activity: no difference between FMS with asymptomatic hypermobility and FMS without asymptomatic hypermobility group (63 vs 61)
Russek L et al. (2014) [38]	Balance confidence: Questionnaires	N/A	N/A	1. Kinesiophobia assessed as >37 on the Tampa Scale of Kinesiophobia	No correlations between the factors and whether they were hypermobile or not. I.e. JHS not discriminatory factor
				2. Joint hypermobility assessed as ≥2 on the Joint Hypermobility Questionnaire	
				3. Balance confidence related to activities was assessed by Activity Balance Confidence Scale.	
Bates AV et al. (2021a) [39]	1. Balance: kinematics and posturography	N/A	N/A	1. Dynamic balance was measured with VICON camera system: kinematics and muscle activity during 6 perturbations from a perturbation platform in AP direction	1. Greater proportion of JHS participants took a recovery step when perturbed; no difference in onset of muscle activities.
	2. Pain: VAS			2. Knee pain was measured with VAS.	2. JHS time-to-peak amplitude of muscle activity was significantly later during the first perturbation than the NF group in Tibialis Anterior (p = .020), Rectus Femoris (p = .002), Vastus Medialis (p = .011), and Vastus Lateralis (p = .002), and significantly later than GJH in Gluteus Maximus (p = .001) and Vastus Lateralis (p = .008). However, in the main it normalised with repeated perturbation.
	3. EMG:			3. Muscle onset and time-to-peak amplitude was measure by EMG	3. No significant difference in time to peak amplitude of muscle activity between GJH and NF groups at any perturbation.
	Muscle onset and time-to-peak amplitude			4. Cumulative sum of CA time-to-reversal	4. Kinematics: Time to reversal of movement was not different between groups. The first perturbation (P1) elicited the greatest magnitude of CA in all groups around all joints. JHS had significantly greater CA than GJH and NF groups. At P1 the group with JHS had significantly greater CA than GJH and NF groups at the hip (p = 0.041 and p = 0.042 respectively) and knee (p = 0.035 and p = 0.009 respectively). At P2 the group with JHS had significantly greater CA than GJH and NF groups at the hip (p = 0.03 and p < 0.01) and knee (p = 0.028 and p < 0.01 respectively), and greater CA at the ankle than NF (p < 0.01). At the final perturbation (P6), the only significant differences were between the group with JHS and NF group, with the group with JHS showing greater hip and knee CA (p = 0.018 and p < 0.01).
	4. Kinematics:				5. No differences in CA between GJH and NF groups at any perturbation number.
	cumulative sum of change in angle (CA)				
	time-to-reversal (TTR)				
Iatridou K et al. (2014) [18]	1.Static postural stability: Posturography	N/A	N/A	1. Static stability on the dominant leg was examined by means of 20-sec single-leg-stance sways with EO and EC; and EO with head extended.	1. Static: ML sway was significantly greater during single-leg-stance with EO (p < 0.01) and EO with head extended (p < 0.05) in the JHS group compared to the control group.
	2. Dynamic stability： Functional test and Vicon camera system			2. AP and ML postural sway was assessed by the vertical (y-component of foot pressure vector) and horizontal (x-component of foot pressure vector) deviation of the centre of foot pressure, using a foot pressure distribution platform (FDMS, Zebris Co., Medical GmbH, Germany)	2. AP sway was greater in the JHS group during single-leg-stance with EO with head extended (p < 0.001).
				3. Dynamic stability was tested by measuring error in landing from multiple single-leg-hops (modified Bass test) on pre-determined markers using a video.	3. Greater number of landing errors during the dynamic test for the JHS group (p < 0.05)
Rigoldi C et al. (2013) [40]	1.Static balance: Posturography and video system	N/A	N/A	Postural sway was measured for 30s while participants stood on a force platform (Kistler, CH; acquisition frequency: 500 Hz) with a fixed position of feet (30° with respect to the AP direction) integrated with a video system whilst EO and EC. Time domain outcomes include: The range of CoP displacement in the AP direction (RANGE AP index) and the ML direction (RANGE ML index), expressed in mm. Trajectory length (TL): the total CoP trajectory length, expressed in mm. All parameters were normalised to the participant's height (expressed in metres). Frequency domain outcomes include: the fast Fourier transform: include the centre frequency of the spectral power peak of the Py spectrum (fy); the centre frequency of the spectral power peak of the Px spectrum (fx).	1. All the participants were able to perform the task without any difficulties.
				Complexity of the patterns of movements within a time domain using more complex algorithms	2. CoP trajectory length didn't differ between groups although AP and ML trajectories longer with EO and EC.
					3. Frequency parameters were not different. There was a change to co-ordination suggesting loss of complexity of movement of hEDS group, but without difference between EO and EC.
Marnili T et al. (2017) [30]	Static Balance and the contribution of proprioception: functional test---single leg stance test	N/A	N/A	Balance time during right and left single leg stance with the EC. Test stopped after 60s or if non-weightbearing leg touched down, participants phopped, or torso or hip bent in compensation to retain balance.	1. No difference in balance time between Hypermobile (median and IQR; 36.50 sec ± 18.8) and non-hypermobile dancers (33.00 ± 18.9, p = 0.982). No correlation between hypermobility and experience.
					2. Dancers demonstrated a higher prevalence of hypermobility compared to that reported for the general population.
Galli M et al. (2011b) [41]	Postural stability Posturography and video system	N/A	N/A	Having discarded the first 10secs of data, the range of CoP displacement in the AP (RANGE AP) and in the ML direction (RANGE ML) (mm) and the trajectory length of CoP (TL) (mm), that was the total CoP trajectory length during quiet stance. All parameters were normalised to the participant's height (m) and to their foot length (mm).	AP (0.09 (0.02) vs 0.02 (0.01)) and ML sway (0.06 (0.03) vs 0.03 (0.02)) and CoP trajectory (4.46 (0.92) vs 0.85 (0.99)) were greater in the hEDS group compared to control. No differences between PWS and hEDS groups.
Galli M et al. (2011a) [42]	Balance: Posturography and video system	N/A	N/A	1. ML COP excursion and the AP COP excursion and trajectory length of the COP.	1. Both ML and AP CoP differed between hEDS with both EO and EC.
				2. Center frequency of the main spectral peak of both AP COP spectrum and ML COP spectrum. Data was collected for 30sec but the first 10s were removed. Data was compared between EO and EC as well as across the two groups	2. CoP was greater with EC in the hEDS group alone.
					3. Trajectory length nor CoP spectrum in AP or ML directions differed across groups or with EO and EC.
Whitmore M et al. (2023) [43]	Force plate	N/A	N/A	COP in hard surface and foam surface; sway velocity; reaction time, movement velocity, endpoint excursion, maximum excursion, and directional control.	1. Significant sway differences were found between various standing conditions which included: EO and EC, hard ground and foam ground, single leg standing and both legs standing,
					2. There was no statistical comparison between hEDS and healthy control groups
Teran-Wodzinski P et al. (2023) [7 ]	1. Pain chart.	N/A	N/A	Author generated questionnaire asking about (pain, fatigue, emotional distress, interference with daily activities, joint instability, hypermobility, reduced proprioception, muscle weakness, walking and balance issues, cardiovascular and gastrointestinal, problems, and orthostatic hypotension etc)	71% reported problems with balance
	2. Questionnaire				
Hou ZC et al. (2023) [44]	Functional tests of balance and strength	Paced progressed balance training from static to dynamic tasks eg single leg stance to hop, plus strengthening programme unclearly defined	N/A	FAAM and ankle sprain recurrence; Star Excursion Balance Test; functional test (Balance error scoring system -double-legged stance, single-legged stance, and tandem stance in a heel-to-toe fashion. Participants performed all stances on firm and foam surfaces (model Balanced; Airex AG, Sins, Switzerland) with their hands on their hips and EC. They performed one practice trial for each condition to ensure proper technique, followed by one test trial. Total errors were counted for each 20-s trial. An error was defined as lifting the hands off the iliac crests; EO; stepping, stumbling, or falling; moving the hip into more than 30° of abduction; lifting the forefoot or heel; or remaining out of test position for more than 5 s) and isokinetic strength at 60 and 120 degrees per second in dorsiflexion/plantarflexion	At 3months post intervention primary outcome of FAAM-S changed more than the minimally clinically important difference and reached a MCID between groups with people with GJH changing more than the non GJH group. Secondary outcomes: 17% of GJH group resprained and 29% of non GJH had resprained. No difference between groups in functional tests. No change or difference between groups in strength, dorsiflexion /plantarflexion improved more in GJH group at 60 degrees per sec, and dorsiflexion alone at 120 degrees per sec.
Hakimi A et al. (2023) [45]	Force plate along with questionnaires, functional tests	9 weeks intervention comprising two-thirds physical activity and one-third educational or mental well-being activities. This was made up of 2 days per week for 4 weeks, then 1 week of rest followed by 3 days per week for final 4 weeks. 4 one-hour workshops including occupational therapy, physiotherapy, sophrology (relaxation alongside meditation and movement), physical activities (included ergometer, hydrotherapy, walking, yoga) focussing on muscular endurance, coordination, balance, and proprioception, or various therapeutic patient education workshops.	N/A	COP, 95% confidence ellipse area encompassing 95% of CoP samples in mm2, and the sway path (mm) over 50 seconds with EO and EC alongside 6-meter walk test and questionnaires such as Multidimensional Fatigue Inventory and Tampa Scale for Kinesiophobia, SF-36, Brief Pain inventory, Nijmegen Questionnaire and HAD	No change in balance after a rehabilitation programme over 6 months. Some changes to balance immediately after the class (COP reduction in EC (mm^2^) changing from 2210±1805 to 1458±1420* and then and at 6 weeks post class (EC 21058±1015 and EO and EC SP changing from 775±284 to 630±173mm and 1478±548 to 1174±487mm respectively
Benistan K et al. (2023b) [46]	Force plate	Application of pressure garments with average pressures ranging from 10 to 15 mmHg. Two sets of 3 pieces (leggings, socks, and vest) worn ≥ 8 h a day for 4 weeks, during activities of daily life and physiotherapy (strengthening, proprioception, and balance exercises, 3X/week)	Physiotherapy: 4-week rehabilitation programme with 1 h of outpatient physiotherapy, 3 times per week, for 4 weeks. Included strengthening the muscles of the ankle, knee, and hip; aerobic exercises; proprioceptive training; and balance exercises on stable and unstable surfaces with EO and EC.	Primary outcome: COP during 8 progressing tasks repeated 3 times until unstable platform introduced when only repeated once: EO on a static firm platform, EC on a static firm platform, EO on a foam surface, EC on a foam surface , EO on an unstable platform that performed AP oscillations, EC on an unstable platform that performed AP oscillations, EO on an unstable platform that performed ML oscillations , EC on an unstable platform that performed ML oscillations. Secondary outcomes were the 90% confidence ellipse area of COP (mm2), Romberg quotient (RQ, RQ = sway area EC / sway area EO) and global joint pain, expressed on the Numerical Pain Rating Scale	Although there were significant differences to postural sway velocity immediately after application of the pressure garment, there were no differences for sway velocity between groups at 4 weeks in the primary outcome except for a reduction in medial-lateral oscillations when with the EC on an unstable platform whilst wearing the pressure garment (intergroup difference of 18.84 mm/s (95% CI 4.36 to 39.23; Effect Size (ES)= 0.93). Of the secondary outcomes there were no differences between intervention groups except for sway area during the same task of 1367mm2 (95% CI 146 to 3274; ES = 0.45) and Romberg quotient with a difference of 0.73 (95% CI 0.09 to 1.69; ES = 0.98)
Benistan K et al. (2023a) [47]	Questionnaires	Compression garments over various sites with 80% wearing socks; 66% wearing trouser length garments	N/A	Berg Balance Scale: 14 tasks rated from 0 to 4 including ability to stand with EC and single leg stance giving total scores from 0 to 56. Scores below 21 indicate that the patient is at high risk of a fall and requires a wheelchair; scores between 21 and 40 indicate that the patient is at medium risk of a fall and requires a walking aid; higher scores indicate that the patient can walk unaided as they are at low risk of a fall.	Related to balance: No change in Berg balance score over 2 years (n dropping to 47).
Bates AV et al. (2021b) [48]	Pain and force plate	N/A	N/A	Sway and fidgets (movements over time)	No difference in number of fidgets between groups. ML sway did not differ between groups although AP sway was greater in the JHS group in comparison to the normal groups with small effect size (p=0.05)

Legend: *Abbreviations:* *N/A* Not applicable, *EDS* Ehlers Danlos Syndromes, *hEDS* hypermobile Ehlers Danlos Syndrome, *GJH* Generalised Joint Hypermobility, *FMS* Fibromyalgia Syndrome, *GJH* Generalised joint hypermobility, *EC* Eyes closed, *EO* Eyes open, *VAS* Visual analogue scale, *COP* Centre of pressure, *FES* Falls efficacy scale, *EMG* Electromyography, *FMS* Fibromyalgia syndrome, *NF* Normal flexibility, *ML* Mediolateral, *AP *Anteroposterior, *CA* Change in angle, *PWS* Prader-Willi Syndrome, *BBS* Berg balance scale, *CI* Confidence interval, *ES* Effect Size, *RQ* Romberg quotient

### Context

Although there were two small randomised controlled trials, ten studies used a case control design and four used a cross-sectional design, thus the level of evidence was low [30, 43, 45, 47]. Eight papers failed to mention the setting of their work; of the rest, the majority (7/11) undertook work within healthcare settings [7, 21, 38, 39, 45–47]. See Table 3 for this detail.

**Table 3:** Aim, setting and design of included papers

Author (year)	Country of Origin	Title	Aim	Setting	Study Design	Level of evidence
Aydın E et al. (2017) [34]	Turkey	Postural balance control in women with generalised joint laxity	To investigate differences in balance using posturography between non-hypermobile, mildly asymptomatic hypermobile and asymptomatic hypermobile participants	Not mentioned	Cross-Sectional	Level 5
Falkerslev S et al. (2013) [35]	Denmark	Dynamic balance during gait in children and adults with Generalised Joint Hypermobility	To investigate if differences of the head and trunk stability and stabilization strategies exist between subjects classified with GJH and healthy controls during gait	Laboratory	Case-Control	Level 4
Toprak Celenay S et al. (2017) [36]	Turkey	Effects of spinal stabilization exercises in women with benign joint hypermobility syndrome: a randomised controlled trial	To investigate the effects of an 8-week lumbar spinal stabilization exercise program on pain, trunk muscle endurance, and postural stability in women with benign JHS	Laboratory	Randomised Controlled Trial	Level 2
Rombaut L et al. (2011) [ 21]	Belgium	Balance, gait, falls, and fear of falling in women with the hypermobility type of Ehlers-Danlos syndrome	To investigate balance, gait, falls, and fear of falling in patients with the hEDS	Primary care	Case-Control	Level 4
Ulus Y et al. (2013) [37]	Turkey	Is there a balance problem in hypermobile patients with fibromyalgia?	To investigate the relationship between hypermobility and balance problem and the possible effect of this relationship on fall frequency in patients with FMS	Not mentioned	Case-Control	Level 4
Russek L et al. (2014) [38]	United States	A cross-sectional survey assessing sources of movement-related fear among people with fibromyalgia syndrome	To assess factors contributing to movement-related fear and to explore relationships among function and wellness in a widespread population of people with FMS	Community setting	Cross-Sectional	Level 5
Bates AV et al. (2021a) [39]	United Kingdom	Adaptation of balance reactions following forward perturbations in people with joint hypermobility syndrome	To compare responses to forward perturbations between people who are hypermobile with joint hypermobility syndrome and without symptoms and people with normal flexibility	Not mentioned	Case-Control	Level 4
Iatridou K et al. (2014) [18]	Greece	Static and dynamic body balance following provocation of the visual and vestibular systems in females with and without joint hypermobility syndrome	To investigate the contribution of proprioception to postural sway, generated with and without challenging the visual and vestibular systems, under static and dynamic conditions in individuals with and without JHS	Not mentioned	Case-Control	Level 4
Rigoldi C et al. (2013) [40]	Italy	Measuring regularity of human postural sway using approximate entropy and sample entropy in patients with Ehlers-Danlos syndrome hypermobility type	To compare postural control for people with hEDS and controls by changing attentional investments in evoking vestibular control using posturography and entropy	Laboratory	Case-Control	Level 4
Marnili T et al. (2017) [30]	United States	Eyes-Closed Single-Limb Balance is Not Related to Hypermobility Status in Dancers	To assess how hypermobility affects EC single-limb stance balance as an indirect measure of proprioception across dancer groups, including student, collegiate, pre-professional, and professional dancers	Not mentioned	Cross-Sectional	Level 5
Galli M et al. (2011b) [41]	Italy	The effects of muscle hypotonia and weakness on balance: A study on Prader-Willi and Ehlers-Danlos syndrome patients	To compare postural control in adult Prader–Willi syndrome and hEDS using platform stabilometry to provide deeper insight into the causes of their postural abnormalities	Primary care	Case-Control	Level 4
Galli M et al. (2011a) [42]	Italy	Postural analysis in time and frequency domains in patients with Ehlers-Danlos syndrome	To evaluate the postural steadiness of hEDS participants and assess the role of proprioception in controlling the standing posture whilst with the EO and EC in time and frequency domain	Laboratory	Case-Control	Level 4
Whitmore M et al. (2023) [43]	United States	A novel method of assessing balance and postural sway in patients with hypermobile Ehlers-Danlos syndrome	To analyse dynamic postural control in people with hEDS	Not mentioned	Cross-Sectional	Level 5
Teran-Wodzinski P et al. (2023) [7 ]	United States	Clinical characteristics of patients with hypermobile type Ehlers-Danlos syndrome (hEDS) and generalised hypermobility spectrum disorders (G-HSD): an online survey	To assess the clinical characteristics of patients with hEDS and G-HSD including areas of concern for people with hypermobility which included balance	Community setting	Cross-Sectional	Level 5
Hou ZC et al. (2023) [44]	China	Balance training benefits chronic ankle instability with generalised joint hypermobility: a prospective cohort study	To investigate whether training balance has a similar outcome for people with chronic ankle instability with and without generalised joint hypermobility	Not mentioned	Case-Control	Level 4
Hakimi A et al. (2023) [45]	France	Multiple Sustainable Benefits of a Rehabilitation Program in Therapeutic Management of Hypermobile Ehlers-Danlos Syndrome: A Prospective and Controlled Study at Short- and Medium-Term	To investigate if a rehabilitation programme for people with hEDS impacts functional exercise capacity, balance, kinesiophobia, pain, fatigue, quality of life, anxiety, depression, and hyperventilation in the short- and medium-term	Primary care	Case-Series	Level 6
Benistan K et al. (2023b) [46]	France	Effects of compression garments on balance in hypermobile Ehlers-Danlos syndrome: a randomised controlled trial	To evaluate the immediate and 4-week effects of wearing compression garments combined with conventional physiotherapy on balance and pain in people with hEDS	Primary care	Randomised Controlled Trial	Level 2
Benistan K et al. (2023a) [47]	France	The Effectiveness of Compression Garments for Reducing Pain in Non-Vascular Ehlers-Danlos Syndromes: A Prospective Observational Cohort Study	To evaluate the effectiveness of compression garments on reducing pain in people with HSD/hEDS after 6 months. Secondary questions were do they change proprioception/balance, joint instability, fatigue, and functional independence over 2 years	Primary and secondary care	Prospective Observational Cohort	Level 3
Bates AV et al. (2021b) [48]	United Kingdom	Prolonged standing behaviour in people with joint hypermobility syndrome	To investigate whether prolonged standing behaviour differs in people with JHS compared to GJH and NF control groups	Not mentioned	Case-Control	Level 4

Legend: *Abbreviations:* *hEDS* hypermobility type of Ehlers-Danlos Syndrome, *GJH* Generalised joint hypermobility, *JHS* Joint hypermobility syndrome, *G-HSD* Generalised hypermobility spectrum disorders, *EDS* Ehlers-Danlos Syndromes, *EO* Eyes open, *EC* Eyes closed, *FMS* Fibromyalgia syndrome

### Findings

For this scoping review, it would be inappropriate to combine data from the papers to form stronger conclusions. However, from the studies included here (see Table 2), authors suggest that sway is increased in people who are hypermobile in comparison to people who are not hypermobile [18, 21, 34, 39, 41, 42]. Further, there is some evidence that people who are symptomatic and hypermobile sway more than people who are asymptomatic and hypermobile [42, 43, 48]. When balance is stressed by removing eyesight or decreasing the stability of the surface, then sway increases further [43]. A key finding is that when the head was placed in extension, a position that impacts both visual, proprioceptive and vestibular systems, sway increased more in symptomatic hypermobile people in comparison to healthy controls [18]. However, it should be noted that one study did not reveal a difference in sway between people who had symptomatic hypermobility and healthy controls [40]. It should also be noted that one study found no correlation between balance and degree of hypermobility measured using the Beighton score [38].

There is little evidence relating to falling or fear of falling, however, two studies surveyed hypermobile people about falling and reported that falling occurs at least once per year in people with hypermobility [21, 37].

## Discussion

To our knowledge, this is the first paper to summarise investigations of balance and falling in people with hypermobility. These investigations suggest that hypermobile people have poorer balance revealed by the increased sway during tasks requiring greater stability such as when vision is disturbed, the vestibular system is perturbed, or the body is placed in positions with a smaller or more unstable base of support. In summary, and importantly for this cohort, the literature presented here suggest that adults who are hypermobile fall and have a fear of falling. It is important to note that an increase in fear of falls can also lead to increased rates of falls [49]. Although current evidence is limited it is interesting to note that when surveyed, 95% of adults with hypermobility who are relatively young, fell in a year [18], whereas UK government guidance on falls suggests that 50% of people aged 80 and above fall in a year [50]. This high rate of falling and fear of falls in a relatively young population is therefore important to understand in order to target interventions effectively to reduce rates of falling. It is important to note that different classification criteria were used to define whether someone was hypermobile, let alone define whether they had symptomatic hypermobility. This could lead to heterogeneity in baseline characteristics including presence or severity of symptoms, which is likely to impact the results on estimation of balance or frequency of falls among this and future cohorts and inaccurate generalisation across all adults with asymptomatic or symptomatic hypermobility. Standardizing diagnostic criteria will be key to reducing heterogeneity, allowing future meta-analysis as well as improving the generalisability of research.

Participants of research should be representative of the cohort from which it is drawn. To identify this, it is vital to ensure that people with protected characteristics such as race and sex are supported to take part in research and therefore are representative of our diverse population [33, 51, 52]. Authors to date have not fully described their cohorts in this way [36, 37, 39–41, 43, 45–47] and future work should report greater demographic detail of their sample in order to reveal how representative they are of the population as a whole. This will increase the trustworthiness of results ensuring conclusions are drawn from samples, which reflect the population. In addition, the work to date has recruited participants from high to upper-middle income countries (see Table 3), further limiting the populations from where the cohorts are recruited [53].

Different factors related to balance have been explored under conditions where greater balance is required. In the main, sway was greater in the majority of people with symptomatic hypermobility compared to healthy controls [18, 21, 34, 42, 43]. However, it is not clear whether this is direction specific or dependent upon task. In prolonged standing only antero-posterior sway was increased in people who were symptomatic with hypermobility compared to asymptomatic hypermobile and non-hypermobile controls [39]. In comparison, where visual and vestibular systems were challenged, both medio-lateral and antero-posterior sway were greater in symptomatic hypermobile people compared to healthy controls [18]. However, even if sway increases differently depending on the methods employed, the outcomes suggest that balance could be impacted by an increase in sway during different tasks for people with symptomatic hypermobility.

It is important to note that if sway is impacted in people who are symptomatic and have hypermobility, this might impact falling. When increased postural sway is recorded in older people, it results in higher risk of falling [54].

Some papers explored the mechanisms that might underpin why people fall. Two studies stressed a person’s proprioception by comparing sway during EO/EC situations and argue that the sway is greater in people who are symptomatic and hypermobile comparing to healthy people [18, 42]. Indeed, other evidence, not explored here, has already suggested that proprioception is poorer in people who are hypermobile, for example, they have a loss of accuracy in mirroring movements and an increased error of repositioning finger position [55, 56]. Another mechanism explored was the vestibular system during quiet standing by extending the neck. They identified that greater sway occurs in such situations in both symptomatic and asymptomatic hypermobile people compared to healthy controls [18, 34].

### Limitation of this review

The outcomes of papers presented in the review should be taken in the context of their case control and cross sectional designs, which reduces their level of evidence (see Table 3). When results from lower level evidence are generalized to reflect the impact on the population of adults with hypermobility, it can lead to significant risks and impacts, such as ineffective or even harmful interventions [57].

Only literature in English was included, leaving the evidence open to inclusion bias as studies conducted in non-English-speaking regions may capture unique findings, cultural contexts, or interventions that are not fully represented in English publications [58]. This might skew the evidence base, as these omitted studies may provide data that either support or counter findings from English-language research [58].

Full papers were not assessed for their risk of bias or quality, however, as a scoping review our aim was to understand the breadth of the evidence rather than the power of the evidence.

### Implications for future research

Although this review has identified falling in this relatively young population as a problem, the evidence base supporting this, and the long-term impacts, needs to improve. More evidence aimed at understanding the mechanisms which might drive this problem will help the development of targeted treatments aimed at reducing risk of falls.

Future investigations should ensure that people from all backgrounds [33] are recruited to powered studies, using standardised classification criteria, making it clear whether participants have symptomatic or asymptomatic hypermobility.

## Conclusions

People who are hypermobile have high incidence of falls and sway more during quiet standing as well as during tasks stressing balance. Although measures of balance have been reported, few papers explore the underlying neurophysiological mechanisms that could lead to an increase in sway and falling, or the relationships between these mechanisms, postural stability and hypermobility.

## Supplementary Information

Supplementary Material 1.

Supplementary Material 2.

## Data Availability

The datasets used and/or analysed during the current study are available from the corresponding author on reasonable request.

## Abbreviations

GJH: Generalised joint hypermobility
JHS: Joint hypermobility syndrome
HSD: Hypermobility Spectrum Disorders
G-HSD: Generalised hypermobility spectrum disorders
EDS: Ehlers-Danlos Syndromes
hEDS: Hypermobility type of Ehlers-Danlos Syndrome
JBI: Joanna Briggs Institute
PPIE: Patient and public involvement and engagement
PCC: Population, concept and context
COP: Centre of pressure
FES: Falls efficacy scale
VAS: Visual analogue scale
EMG: Electromyography
FMS: Fibromyalgia syndrome
NF: Normal flexibility
ML: Mediolateral
AP: Anteroposterior
CA: Change in angle
EO: Eyes open
EC: Eyes closed
PWS: Prader-Willi Syndrome
BBS: Berg balance scale
CI: Confidence interval
ES: Effect Size
RQ: Romberg quotient
