# Supplementary material for: Falls and balance impairment; what and how has this been measured in adults with joint hypermobility? A scoping review
Source: BMC Musculoskelet Disord. 2025 Jan 28;26:88. doi: 10.1186/s12891-025-08318-3 (PMC11773773; doi:10.1186/s12891-025-08318-3)
Supplement: Supplementary file 1 — Supplementary Material 1. [file 12891_2025_8318_MOESM1_ESM.docx]

Keywords and search strategy

1. Joint Instability
2. hypermobil*.mp.
3. (ehlers-danlos or hEDS).mp
4. Ehlers-Danlos Syndrome/
5. 1 or 2 or 3 or 4
6. Accidental Falls/pc, sn, td
7. Postural Balance/is, ph, pp, px
8. balance*.mp.
9. fell.mp.
10. (slip* or trip* or stumbl* or tumbl*).mp.
11. 6 or 7 or 8 or 9 or 10
12. Electromyography/cl, is, mt, td
13. (Electromyograph* or EMG).mp.
14. Electrooculography/cl, is, mt, td
15. (Electrooculograph* or EOG).mp.
16. Vestibular Diseases/cl, co, di, dg, et, pa, pp, pc, px, rh, su, th
17. posturograph*.mp.
18. (MMG or Mechanical myography).mp.
19. exp Vestibular Function Tests/cl, is, mt, px, td, st
20. caloric test*.mp.
21. electronystagmograph*.mp.
22. head impulse test*.mp.
23. vestibul*.mp.
24. Reflex, Vestibulo-Ocular/ph
25. Eye Movements/di, is, mt, ph, pp
26. (videonystagmograph* or VNG).mp.
27. (rotary adj2 test*).mp.
28. Dix hallpike maneuver.mp.
29. Postural Orthostatic Tachycardia Syndrome/cl, di, dg, ep, et, pa, pp, co, pc, px, rh, th
30. (Postural adj2 Tachycardia).mp.
31. force plat*.mp.
32. ((Time* Up adj2 Go) or TUG).mp.
33. Chair Stand Test*.mp.
34. 4-Stage Balanc*.mp.
35. (5XSST or 5-times sit-to stand).mp.
36. (10MWT or 10-met* walk).mp.
37. berg balance.mp.
38. Biodex Balance.mp.
39. (single leg stan* or single limb stan*).mp.
40. perturba*.mp.
41. elastrograph*.mp.
42. sway*.mp.
43. cent* of gravit*.mp.
44. star excursion.mp.
45. Proprioception/cl, ph
46. propriocep*.mp.
47. joint* position* sense*.mp.
48. kine*.mp.
49. 12 or 13 or 14 or 15 or 16 or 17 or 18 or 19 or 20 or 21 or 22 or 23 or 24 or 25 or 26 or 27 or 28 or 29 or 30 or 31 or 32 or 33 or 34 or 35 or 36 or 37 or 38 or 39 or 40 or 41 or 42 or 43 or 44 or 45 or 46 or 47 or 48
50. 5 AND 11 AND 49
51. yr="2023-Current" (final search was repeated but limited to 2023 to assess for any additional papers published since initial search using the same strategy but with a limit to yr="2023 -Current")
52. 5 AND 11 AND 49 AND 51
